# Population Pharmacokinetic Analysis of Perampanel in Portuguese Patients Diagnosed with Refractory Epilepsy

**DOI:** 10.3390/pharmaceutics15061704

**Published:** 2023-06-10

**Authors:** Rui Silva, Helena Colom, Joana Bicker, Anabela Almeida, Ana Silva, Francisco Sales, Isabel Santana, Amílcar Falcão, Ana Fortuna

**Affiliations:** 1 Laboratory of Pharmacology, Faculty of Pharmacy, University of Coimbra, 3000-548 Coimbra, Portugal; rui.freixo@gmail.com (R.S.);; 2 CIBIT/ICNAS—Coimbra Institute for Biomedical Imaging and Translational Research, University of Coimbra, 3000-548 Coimbra, Portugal; almeida.anabela@gmail.com; 3 Farmacoteràpia, Farmacogenètica i Tecnologia Farmacèutica, IDIBELL—Institut d’Investigació Biomèdica de Bellvitge, 08907 Hospitalet de Llobregat, Spain; helena.colom@ub.edu; 4 Pharmacy and Pharmaceutical Technology and Physical Chemistry Department, Universitat de Barcelona, 08028 Barcelona, Spain; 5 CIVG—Vasco da Gama Research Center, EUVG—Vasco da Gama University School, 3020-210 Coimbra, Portugal; 6 Refractory Epilepsy Reference Centre, Centro Hospitalar e Universitário de Coimbra, EPE, 3004-561 Coimbra, Portugalfranciscosales@chuc.min-saude.pt (F.S.);

**Keywords:** perampanel, epilepsy, population pharmacokinetics, NONMEM, therapeutic drug monitoring

## Abstract

Perampanel is a promising antiepileptic drug (AED) for refractory epilepsy treatment due to its innovative mechanism of action. This study aimed to develop a population pharmacokinetic (PopPK) model to be further used in initial dose optimization of perampanel in patients diagnosed with refractory epilepsy. A total of seventy-two plasma concentrations of perampanel obtained from forty-four patients were analyzed through a population pharmacokinetic approach by means of nonlinear mixed effects modeling (NONMEM). A one-compartment model with first-order elimination best described the pharmacokinetic profiles of perampanel. Interpatient variability (IPV) was entered on clearance (CL), while the residual error (RE) was modeled as proportional. The presence of enzyme-inducing AEDs (EIAEDs) and body mass index (BMI) were found as significant covariates for CL and volume of distribution (V), respectively. The mean (relative standard error) estimates for CL and V of the final model were 0.419 L/h (5.56%) and 29.50 (6.41%), respectively. IPV was 30.84% and the proportional RE was 6.44%. Internal validation demonstrated an acceptable predictive performance of the final model. A reliable population pharmacokinetic model was successfully developed, and it is the first enrolling real-life adults diagnosed with refractory epilepsy.

## 1. Introduction

Characterized by recurrent and unpredictable interruptions of normal brain function, epilepsy is an heterogeneous group of complex diseases and one of the most common neurological disorders worldwide [1,2]. The main therapeutic approach clinically applied for epileptic seizure control is pharmacological treatment with one or more antiepileptic drugs (AEDs) that restore the balance between cerebral excitation and inhibition through several pharmacological mechanisms, such as modulation of voltage-gated ion channels, potentiation of GABAergic activity, inhibition of glutamatergic processes, and modification of the neurotransmitter release [3,4,5]. Although epilepsy is one of the oldest diseases and more than twenty AEDs are currently used in clinical practice, one-third of adequately medicated patients remain with uncontrolled seizures, requiring a greater social interest and increasing awareness of epilepsy [6,7]. AEDs with innovative mechanisms of action seem to be a promising choice when other AEDs fail. However, they remain unable to provide an effective treatment in all patients with refractory epilepsy [8,9].

Personalizing the dose scheme for each patient in an attempt to improve the efficacy and tolerability of AEDs is hence emergent [10,11]. In this context, therapeutic drug monitoring (TDM) and population pharmacokinetic (PopPK) models are prominent decision-making support tools for the definition and optimization of pharmacological therapy through the design of pharmacotherapeutic schemes according to the specific characteristics of each patient [12,13,14]. Particularly, they have been applied to individualize AED dosing and promote seizure control without adverse effects that significantly affect the patient’s quality of life [10,11,15,16,17,18]. TDM ensures optimal exposure to avoid treatment failures resulting from low concentrations and side effects that result from concentrations above the therapeutic window. Indeed, TDM is a clinical tool applied to improve drug efficacy through the individual adjustment of therapeutic dosing regimens. This has been applied in classic drugs, such as phenytoin or digoxin [19], as well as new drugs, such as tyrosine kinase inhibitors [20] or direct oral anticoagulants [21]. On the other hand, PopPK modeling has been ascribed as a robust tool for precision medication in comparison to classic pharmacokinetic analysis, which needs a complete set of concentrations at each time point [13,14,22]. Their combination quantitatively assesses the pharmacokinetic characteristics and interpatient variability (IPV), contributing to a personalized/precise pharmacotherapy. Their application is straightforward in drugs with narrow therapeutic range, IPV and when there is a relationship between drug pharmacokinetics and pharmacodynamics [23]. 

Perampanel is a third-generation AED, approved as adjunctive treatment of focal-onset seizures with or without secondarily generalization and for primary generalized tonic-clonic seizures in patients with idiopathic generalized epilepsy [24]. It meets the previously mentioned three criteria, making the development of PopPK models necessary to integrate TDM in clinical practice and individualize drug posology. Indeed, the efficacy and tolerability of perampanel are directly related to its systemic exposure. Based on a clinical study enrolling pharmacoresistant epileptic patients with focal-onset seizures [25], the narrow therapeutic window of perampanel was established by the International League Against Epilepsy as 0.180–0.980 mg/L [16]. However, a wider range of 0.1–1.0 mg/L has been applied until more clinical information is available [26]. Furthermore, multiple factors determine plasma drug exposure, resulting in large IPV of pharmacological effects. In spite of its rapid and complete intestinal absorption (bioavailability ≈ 100%) [27], perampanel is bound to plasma proteins, mainly albumin and α-1-acid glycoprotein, in the range between 95 and 97% [27,28]. Additionally, its volume of distribution is approximately 1.1 L/70 kg and it is extensively metabolized primarily by the cytochrome P450 3A4 (CYP3A4) isoenzyme, leading to various pharmacologically inactive metabolites [27]. These characteristics increase drug–drug interaction potential and the variability of its response. 

To the best of our knowledge, there are currently only two published PopPK perampanel models, constructed with data from phase II and phase III clinical trials [29,30]. One enrolls adolescents, and none include patients with refractory epilepsy. Therefore, while covering every step of a modeling and simulation workflow, the present investigation aimed to develop a PopPK model to be further used in initial dose optimization of perampanel in adults diagnosed with refractory epilepsy. 

## 2. Materials and Methods

### 2.1. Patients and Pharmacotherapy

A retrospective observational study was performed including 44 Portuguese refractory epileptic patients admitted to the Refractory Epilepsy Centre of the Coimbra Hospital and University Centre, EPE (CHUC, EPE, Coimbra, Portugal) between April 2019 and December 2022. The study was approved by the Ethics Committee of the Faculty of Medicine of the University of Coimbra, Coimbra, Portugal (CE-061/2018) and by the Ethics Committee of CHUC, EPE (CHUC-144-18). The anonymity of all patients was ensured. Inclusion criteria were: patients older than 18 years of age, diagnosis of refractory epilepsy, and perampanel treatment for seizure control for at least a month and submitted to TDM as part of their routine clinical management. Patients were considered refractory when two appropriate and tolerated AED schedules failed to achieve sustained seizure freedom [31]. Patients chronically prescribed with drugs other than AEDs were excluded, as well as those admitted to the intensive care unit or diagnosed with *status epilepticus*. The following data were collected: sex, age (years), weight (kg), height (cm), and the prescribed AED regimen (including drugs and respective posology). Body surface area (BSA, m^2^) and body mass index (BMI, kg/m^2^) were calculated.

### 2.2. Laboratory Testing

Blood samples were collected in tubes containing a clot activator and serum gel separator in order to quantify albumin (g/dL), total proteins (g/dL), alanine aminotransferase (U/L), alkaline phosphatase (U/L), aspartate aminotransferase (U/L), gamma-glutamyl transferase (U/L), lactate dehydrogenase (U/L), total bilirubin (mg/dL), C-reactive protein (mg/dL), and serum creatinine (mg/dL). Serum concentrations were determined through the integrated clinical chemistry and immunoassay equipment Alinity ci-series (Abbott Diagnostics). Glomerular filtration rate (eGFR, mL/min/1.73 m^2^) was estimated by the Chronic Kidney Disease Epidemiology Collaboration equations [32].

### 2.3. Blood Sampling and Perampanel Quantification

A total of 72 plasma concentrations of perampanel were obtained at steady-state. Blood sample collection was performed according to the TDM protocol implemented in the Refractory Epilepsy Centre of CHUC, EPE. Since perampanel is once daily administered and at bedtime, blood samples were collected in the following morning (from 9.7 to 14 h after drug intake, *n* = 42) and before drug administration (from 20.5 to 24 h after drug intake, *n* = 30). Blood sampling was performed while avoiding meal times. The date and time of each sample collection were recorded. Blood samples were collected in heparin-lithium tubes and centrifuged at 4 °C and 2880 *g* for 10 min, followed by plasma collection for further sample treatment and drug quantification. Plasma samples were submitted to a double liquid-liquid extraction procedure with ethyl acetate and drug concentrations were determined by a validated high-performance liquid chromatography method with diode array detection, as previously described in Sabença et al. [33]. The method was linear in the concentration range of 0.03 to 4.50 mg/L.

### 2.4. Population Pharmacokinetic Analysis

Perampanel plasma concentration time data were analyzed through the PopPK approach by means of nonlinear mixed effects modeling resorting to the software NONMEM^®^ version 7.4 (ICON Early Phase, San Antonio, TX, USA). The first-order conditional estimation method with interaction (FOCEI) was used for the parameter estimation and model construction process.

#### 2.4.1. Base Model Development

One- and two-compartment with first-order elimination models were firstly used to fit the concentration-time data of perampanel. Since concentrations were obtained during the elimination phase, the absorption rate constant of perampanel could not be determined and, hence, the intravenous bolus administration was herein chosen, similarly to other published investigations [29,30]. IPV was evaluated for all pharmacokinetic parameters and modeled exponentially, assuming a log-normal distribution. Additive, proportional, and combined (additive + proportional) errors were tested and compared to assess the residual error (RE) associated with drug concentrations. The log-likelihood ratio test was used to compare nested models. A significance level of *p*-value < 0.005 corresponding to a difference in the minimum objective function value of 7.88 was adopted [34,35]. The physiological plausibility of the model parameters and its estimation precision, expressed as the relative standard error (RSE), were also considered.

#### 2.4.2. Covariate Model Development

Demographic (gender, age), anthropometric (weight, height, BSA, BMI), clinical parameters (albumin, total proteins, alanine aminotransferase, alkaline phosphatase, aspartate aminotransferase, gamma-glutamyl transferase, lactate dehydrogenase and total bilirubin), and renal function (given by eGFR) were tested for inclusion as covariates in the model. Moreover, the influence of each concomitant AED was investigated as well as the impact of enzyme-inducing AEDs (EIAEDs) that include carbamazepine, oxcarbazepine, phenobarbital, and phenytoin, and the non-EIAEDs, which include clobazam, eslicarbazepine acetate, lacosamide, lamotrigine, levetiracetam, topiramate, valproic acid and zonisamide).

Firstly, covariates were investigated by a univariate approach, and secondly, by stepwise forward inclusion and stepwise backward elimination procedures. The effect of the inclusion of each covariate on the pharmacokinetic parameters of perampanel was considered significant when the minimum objective function value (MOFV) decreased by at least 3.84 (*p* = 0.05). The included covariates were retained if its elimination resulted in an increase in MOFV of at least 10.8 (*p* = 0.001) [34,35]. Furthermore, the inclusion of each covariate was also evaluated according to (i) the physiological plausibility of the pharmacokinetic parameter values, which had to be similar to the previous analysis, (ii) the precision of estimated parameters, expressed as RSE, (iii) significant clinical reduction in IPV associated to each pharmacokinetic parameter (10%), (iv) condition number, which is the square root of the ratio of the major to the minor eigenvalue, (v) the ƞ- and Ɛ-shrinkage values, as measures of model overparameterization [36], and (vi) the visual inspection of the goodness-of-fit plots which included: observed concentrations (OBS) versus typical population model predicted (PRED) or individual predicted concentrations (IPRED), individual weighted residuals (IWRES) versus IPRED and conditional weighted residuals (CWRES) versus time [37,38].

#### 2.4.3. Model Evaluation

Internal validation of the final model was assessed using prediction-corrected visual predictive check (VPC) [39] and bootstrap methods [40,41,42]. Based on 1000 replicated data sets from the original data set, prediction-corrected VPC was assessed to investigate the predictive ability of the method. Median, 2.5th and 97.5th percentiles of the observations were checked whether they were within the non-parametric 95% confidence intervals of the median, 2.5th and 97.5th percentiles of the simulated profiles. The stability and precision of the final model were evaluated resorting to bootstrap method, using 50 resamplings from the initial data set. The fixed and random effect parameters were calculated as median and 95% confidence interval (2.5% and 97.5% percentiles). For each parameter, bias was determined as the percentage of the difference between median derived from bootstrap and the final population estimate. 

### 2.5. Model-Based Simulations

From the final model, Monte Carlo simulations were carried out with NONMEM^®^ software version 7.4 to evaluate the effect of the covariate combination in the relationship between perampanel dose and its plasma concentrations. In the present study, we found that BMI and concomitant EIAEDs affected perampanel clearance. Therefore, concentration-time profiles for different perampanel dosing regimens (2, 4, 6, 8, 10 and 12 mg once daily) were simulated according to BMI (18.5, 22.5, 27.5 and 32.5 kg/m^2^) and the presence or absence of EIAEDs.

In addition, concentration-time profiles for the same daily doses of perampanel were divided in one or two daily administrations and simulated according to BMI in the presence of EIAEDs. Each simulation generated 1000 perampanel concentration-time profiles for of each combination of covariates using the final estimates of V and CL. Mean trough plasma concentrations were calculated for each combination of covariates. Considering the therapeutic plasma range for perampanel (0.1 to 1.0 mg/mL), trough plasma concentrations were classified as under, within and over the therapeutic range and compared between the several groups.

### 2.6. Statistical Analysis

R software version 4.1.3 (The R Foundation for Statistical Computing) was used for statistical analysis and nonlinear mixed effects model diagnostics. Descriptive statistics were stated as absolute and relative frequencies for categorical variables and as median (minimum–maximum) for continuous variables. The package xpose4 version 4.7.1 was used to guide the model building.

## 3. Results

A population pharmacokinetic model was developed resorting to seventy-two plasma concentrations of perampanel obtained from forty-four refractory epileptic patients. Patient characteristics are described in Table 1.

Most of the patients were men (61.4%) with a median of 38 years of age (19–76), while 38.60% were women with a median of 32 years of age (21–67). The observed median BMI in men (25.10 kg/m^2^) and women (26.22 kg/m^2^) corresponds to the overweight category. With the exception of alkaline phosphatase and gamma-glutamyl transferase, remaining clinical results were all within normal clinical ranges (Table 1). Median eGFR was 110.63 mL/min/1.73 m^2^; however, it ranged from moderately decreased to normal values (49.31–134.90 mL/min/1.73 m^2^). Perampanel dosing regimens ranged from 2 to 10 mg daily, although the most frequently administered daily doses ranged from 4 to 8 mg.

It should be noted that most patients were on polytherapy with two (36.4%) and three (29.5%) AEDs besides perampanel. Levetiracetam (63.6%) and carbamazepine (34.1%) were the most often co-prescribed AEDs. Twenty (45.5%) patients were co-prescribed with EIAEDs. More details can be seen in Table S1 (Supplementary Materials).

### 3.1. Population Pharmacokinetic Modeling

Plasma concentrations of perampanel were better described by a one-compartment model with first-order elimination parameterized by volume of distribution (V) and clearance (CL) than by a two-compartment model. IPV was entered on CL, while residual variability was modelled as proportional.

Inclusion of all the candidate covariates in the model, one at each step, identified BW, BSA, and BMI as covariates with statistically significant effects on V, and gamma-glutamyl transferase, the presence of carbamazepine, clobazam, lacosamide, phenytoin, and the group of EIAEDs as covariates significantly affecting the CL (Supplementary Materials: Tables S2–S5).

The final covariate model only retained the presence of EIAEDs and BMI as covariates for CL and V, respectively. Inclusion of EIAEDs resulted in a decrease in MOFV of −54.259 and IPV of −27.8%. The sequential inclusion of BMI in V resulted in a decrease in MOFV of −19.965. The proportional residual error decreased from 9.03% in the base model to 6.44% in the final covariate model.

Thus, in the final model, the presence of EIAEDs, which included carbamazepine, oxcarbazepine, phenobarbital, and phenytoin, increased the typical value of perampanel CL 2.76-times. BMI normalized by its median value affected the V of perampanel directly and positively. The final model equations were as follows in Equations (1) and (2):

(1) $$V(L)=29.5\times {\left(\frac{BMI}{25.1}\right)}^{2.12}$$

(2) $$CL(L/h)=0.419\times 2.76^{IND}$$

in which, BMI is the body mass index, in kg/m^2^, and IND is equal to 0 or 1 if EIAEDs are absent or present.

Parameter estimates of the base and final models as well as the bootstrap results are summarized in Table 2. The successful ratio of minimization runs was one. All pharmacokinetic parameters were estimated with adequate precision. Mean population values of model parameters were within the 95% confidence intervals estimated by the bootstrap method. The relative deviation between the true population value and the median value provided by bootstrap was lower than 10% for all pharmacokinetic parameters. The condition number of the model was 2.63, suggesting no notable collinearity. Acceptable values were found for the Ɛ-shrinkage (36.1%) and ƞ-shrinkage (0.01%) associated to the CL.

**Figure 1:**
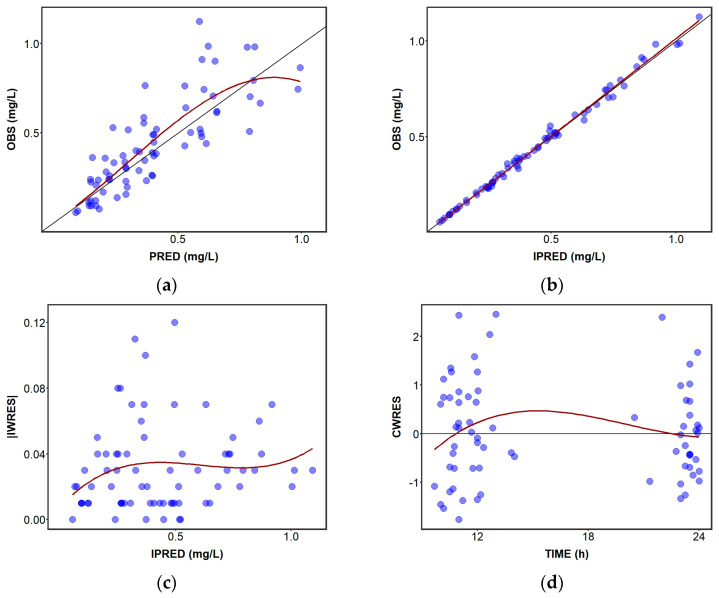
Goodness-of fit plots of the final model: (**a**) population predicted (PRED) concentrations vs. observed (OBS) concentrations, (**b**) individual predicted concentrations (IPRED) vs. OBS concentrations, (**c**) absolute individual weighted residuals (IWRES) vs. individual predicted concentrations, and (**d**) conditional weighted residuals (CWRES) vs. time after dose. OBS, observed concentrations (mg/L); IPRED, individual predicted concentrations (mg/L); PRED, population predicted concentrations (mg/L). Blue dots represent perampanel plasma concentrations; Black line, line of identity; Red line, data smoother.

The main goodness-of-fit plots for the final model are depicted in Figure 1. The OBS concentration versus PRED concentrations spread randomly around the identity line (Figure 1a), while conditional weighted residuals (CWRES) vs. time (Figure 1d) uniformly spread around the zero line (Figure 1c), indicating no model misspecification. Furthermore, OBS concentrations versus IPRED concentrations scattered around the identity line (Figure 1b) as well as IWRES versus IPRED uniformly spread around zero (Figure 1c), suggesting an adequate description of IPV and RE, respectively.

**Figure 2:**
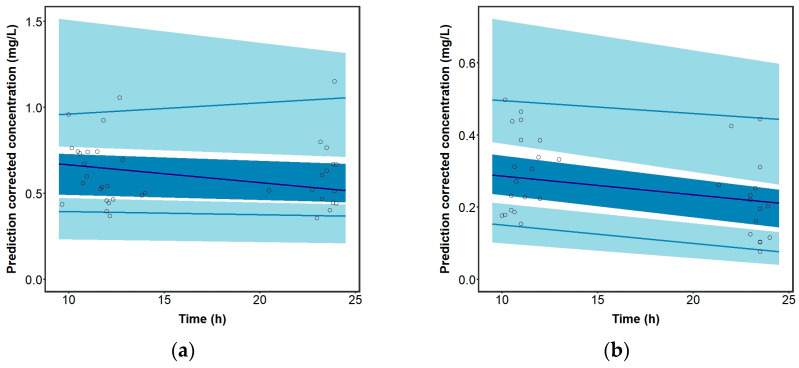
Prediction-corrected visual predictive check of the final model for (**a**) patients not taking and (**b**) patients taking EIAEDs. The dots represent the prediction-corrected concentrations of perampanel (mg/L) at the respective time after dose administration. The lines represent from top to bottom the 2.5th, 50th and 97.5th observed percentiles, respectively. Dark blue shading displays the simulated based 95% confidence intervals for the 50th percentile and light blue shading the 2.5th and 97.5th percentiles.

Prediction-corrected VPC indicated that the final model could acceptably predict the distribution of the observed plasma concentrations of perampanel for patients not co-administered (Figure 2a) and administered EIAEDs (Figure 2b). The parameter estimates obtained with the final model are consistent with the median parameter estimates from the bootstrap and are within the 95% confidence intervals (Table 2).

### 3.2. Model-Based Dosing Simulations

Table 3 displays the mean steady-state trough plasma concentrations of perampanel simulated for each daily dose according to BMI, and with or without EIAED co-administration. For each daily dose, mean trough plasma concentrations increased with BMI, and were higher in patients not co-administered with EIAEDs (0.151 to 1.145 mg/L) than in co-administered patients (0.030 to 0.347 mg/L).

In patients not co-administered with EIAEDs, mean steady-state trough plasma concentrations were within the therapeutic range (0.1 to 1.0 mg/L) when perampanel doses ranged from 2 to 10 mg/daily. When the dose was 12 mg/daily, mean steady-state trough plasma concentration remained within the therapeutic range only for the subpopulation with a BMI of 18.5 kg/m^2^. In contrast, patients co-medicated with EIAEDs revealed mean steady-state trough plasma concentrations within the therapeutic range only when the dose of perampanel ranged from 6 to 12 mg/daily (Table 3). Doses of 2 and 4 mg/day revealed concentrations bellow the therapeutic range (<0.1 mg/L).

**Figure 3:**
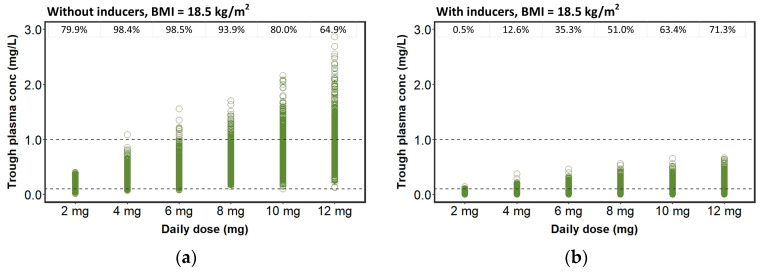

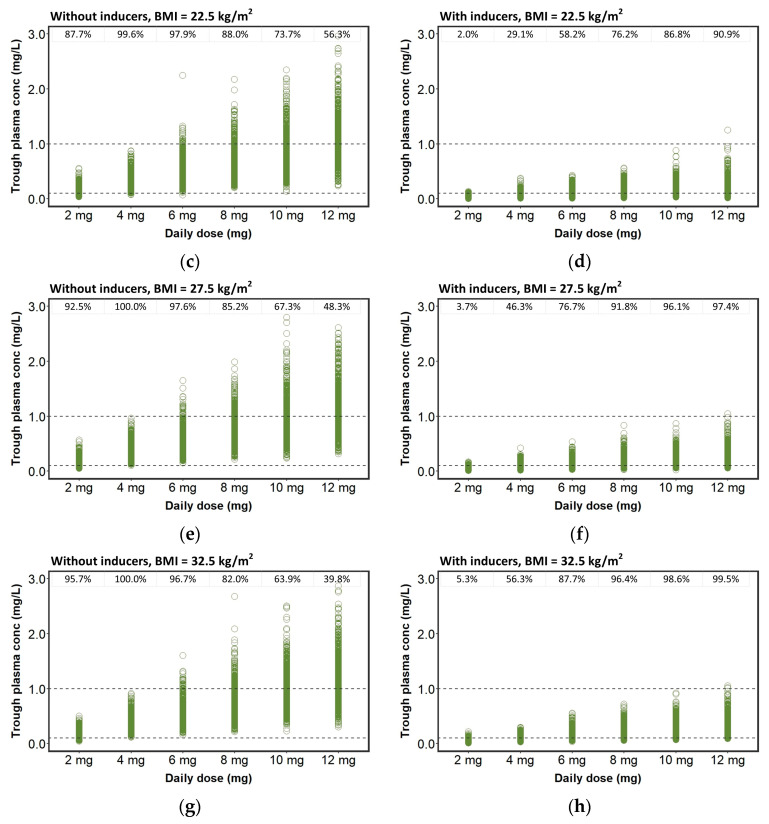
Plasma concentrations (mg/L) simulated for once-daily perampanel in patients not taking EIAEDs and (**a**) body mass index (BMI) = 18.5 kg/m^2^, (**c**) BMI = 22.5 kg/m^2^, (**e**) BMI = 27.5 kg/m^2^ and (**g**) BMI = 32.5 kg/m^2^ and in patients taking EIAEDs and (**b**) BMI = 18.5 kg/m^2^, (**d**) BMI = 22.5 kg/m^2^, (**f**) BMI = 27.5 kg/m^2^ and (**h**) BMI = 32.5 kg/m^2^. The percentages of perampanel plasma concentrations within the therapeutic range (0.1–1.0 mg/L) are displayed on top of the graph.

Figure 3 depicts the percentages of steady-state trough plasma concentrations simulated from different daily doses of perampanel within and outside the therapeutic range according to BMI and EIAED co-administration. Regarding patients not taking EIAEDs, daily doses ranging from 4 mg to 8 mg provided the highest proportions (>90%) of trough plasma concentrations within the therapeutic range. Significant percentages of trough plasma concentrations above the therapeutic range (>20%) were found among patients with daily doses of 10 and 12 mg. Considering patients co-medicated with EIAEDs, proportions higher than >90% were found in patients treated with perampanel doses of 8 to 12 mg/daily. Significant percentages (>20%) of trough plasma concentrations under the therapeutic range were found among patients under 2 and 8 mg/daily.

**Figure 4:**
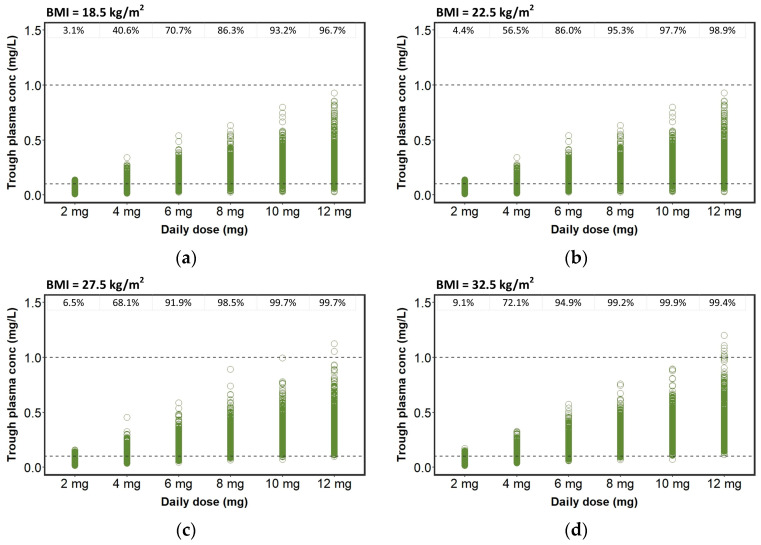
Plasma concentrations (mg/L) simulated for twice-daily perampanel in patients taking EIAEDs and (**a**) body mass index (BMI) = 18.5 kg/m^2^, (**b**) BMI = 22.5 kg/m^2^, (**c**) BMI = 27.5 kg/m^2^ and (**d**) BMI = 32.5 kg/m^2^. The percentages of perampanel plasma concentrations within the therapeutic range (0.1–1.0 mg/L) are displayed on top of the graph.

Due to the low trough plasma concentrations of perampanel observed in patients taking EIAEDs, a new set of simulations were performed considering a twice daily administration and the corresponding mean steady-state trough plasma concentrations are summarized in Table 4, while the percentages of steady-state trough plasma concentrations, within and outside the therapeutic range, are depicted in Figure 4.

## 4. Discussion

Personalized medicine in pharmacotherapy, although not a new concept, is currently gaining interest not only by the medical community and other health professionals, but also by the scientific community and academic institutions [43]. TDM together with PopPK accurately support the design of pharmacotherapeutic schemes according to the specific characteristics of each patient and their pathological state. Therefore, model-informed precision dosing is an a priori TDM strategy based on the application of mathematical and statistical models developed for specific populations of patients. Furthermore, these models could be employed through a Bayesian approach to adjust dosing regimens a posteriori [14,44].

A PopPK model for perampanel was herein successfully developed in a population of Portuguese patients diagnosed with refractory epilepsy. There are currently two other PopPK models for perampanel; however, the model herein reported is the only model that includes data from adult refractory epileptic patients under real TDM processes instead of patients enrolled in clinical trials [29,30]. 

Consistent with the sparse nature of the data collected during the elimination phase and in accordance with the previous studies [29,30], the pharmacokinetics of perampanel in the present population was best described by a one-compartment model with first-order elimination. Herein, the typical estimated CL (0.419 L/h) for perampanel was approximately 50–60% lower than the typical CL estimated by Villanueva et al. [29] (0.729 L/h) and Takenaka et al. [30] (0.668 L/h). In the cited studies, it was not possible to estimate the V of perampanel and therefore it was fixed at 43.5 L. Herein, clinical data allowed an accurate estimation of V for perampanel (29.5 L), which is lower than those used in the literature (43.5 L). This can be attributed to the fact that the plasma concentrations were obtained after the distribution phase (9.7 to 24 h after drug intake). Importantly, we found lower proportional residual error (6.44%) and IPV associated to CL (30.82%) comparatively to already published models [29,30]. These findings highlight that the present model is clinically relevant and can be further used in drug individualization. Indeed, due the small residual variability herein observed, the overall expected variation is mainly associated with CL, which is overcome when BMI and inducers are included to individualize the posology of perampanel.

To identify the factors affecting the pharmacokinetics of perampanel, several covariates were tested for inclusion in the model. In agreement with already published models [29,30], age did not affect the pharmacokinetic parameters of perampanel in our study. On the other hand, the same authors observed a significant difference between the CL of male and female patients, but in the real-life study presented by Patsalos et al. [45] there were no differences between perampanel pharmacokinetic parameters in women and men. Similarly, in our study, gender did not affect the pharmacokinetic parameters of perampanel. None of the analytical variables herein investigated revealed a significant impact on perampanel pharmacokinetics, probably because these characteristics were within normal clinical ranges for most patients (Table 1, Section 3). Ideally, patients with values out of the reference range should also be included and investigated. 

The co-administration of EIAEDs and BMI were found as significant covariates that affect the CL and V of perampanel, respectively. The co-administration of EIAEDs increased the typical value of perampanel CL by two and seventy-six hundredths-fold, in accordance with reported models [29,30]. Indeed, it is well-known that perampanel is eliminated by hepatic CYP450 isoenzymes, mainly CYP3A4, and CYP3A5 to a minor extent [27,46,47]. Genetic polymorphisms in CYP3A4 do not influence perampanel CL [48]. However, EIAEDs enhance the metabolism of perampanel. Given that perampanel is approved as adjunctive therapy, co-prescription with EIAEDs may decrease its plasma concentrations, probably compromising drug efficacy [45,49]. The magnitude of the inducing effect of each of the EIAEDs has been reported to be different and dose dependent [27,45,49]. A study including data from phase III clinical trials showed that carbamazepine was the drug with the greatest inducing effect, causing a reduction of 67% in the area under the concentration-time curve of perampanel, compared with a reduction of 50% caused by oxcarbazepine and phenytoin [27]. These AEDs, particularly carbamazepine, continue to be used with considerable frequency in clinical practice [50].

In the present model, EIAEDs demonstrated the greatest impact on perampanel CL comparatively to each EIAEDs individually, probably due to the small sample when considering each EIAED and combination of EIAEDs. However, previous models [29,30] were developed resorting to large populations, whereby, it was possible to identify the effects of EIAEDs, separately. Carbamazepine was the main EIAED affecting the CL of perampanel, followed by oxcarbazepine/phenytoin. Carbamazepine is the EIAED with the highest effect, augmenting the CL of perampanel by two and sixty-four hundredths-fold [29] and two and ninety-five hundredths-fold [30]. The presence of oxcarbazepine/phenytoin showed an increase of one and seventy-eight hundredths-fold [29] and one and ninety-nine hundredths-fold [30] and topiramate/phenobarbital of one and twenty-one hundredths-fold [30].

On the other hand, BMI was identified as an individual characteristic that affected the V of perampanel in a direct relationship, probably because the extracellular water increases as the fat mass enhances [51] and the V of perampanel is closer to total body water [27]. Indeed, the patients herein enrolled encompassed a wide range of BMI (from 15.76 to 36.10 kg/m^2^), allowing its association with V, which is a pharmacokinetic parameter useful for drug load definition. Regarding the effect of weight on perampanel pharmacokinetics, only Yamamoto et al. [49] suggested that, in addition to the effect of EIAEDs, age, sex, body weight, and CYP3A5 polymorphisms may contribute to fluctuations of perampanel plasma concentrations.

Importantly, the results found during the model internal validation showed an acceptable predictive performance (Table 2 and Figure 2), supporting its use in clinical practice to design effective and safe dose regimens of perampanel in patients diagnosed with refractory epilepsy. Thus, this model can be applied not only to design a priori dosing regimens, but also to design a posteriori adjustments of perampanel dosing regimens, employing a Bayesian approach.

After model development and validation, it was used to perform model-based simulations aiming to define the maintenance dosing posology of perampanel in patients diagnosed with refractory epilepsy. According to the summary of product characteristics, the maintenance dose of perampanel recommended for adults is 4 mg to 8 mg daily for patients diagnosed with focal-onset seizures, and up to 8 mg daily for patients diagnosed with primary generalized tonic-clonic seizures. In both cases, the maintenance dose must be titrated from 2 mg daily to the recommended dose, according to patient individual response. The drug should be taken orally, once daily and at bedtime to minimize its most common adverse events, namely dizziness, somnolence, and fatigue [24].

The efficacy and tolerability of perampanel is directly related with its systemic exposure [25]. Thus, applying the developed model, simulations of concentration-time profiles of perampanel were performed for doses ranging from 2 mg to 12 mg according to various scenarios, combining the absence or presence of EIAEDs with BMI. Then, trough plasma concentrations were evaluated according to the therapeutic range established for perampanel (0.1 to 1.0 mg/L). As V affects mainly the maximum concentrations, a great effect of BMI on trough plasma concentrations was not expected. 

More than 90% of the patients not co-administered with EIAEDs were expected to exhibit trough plasma concentrations within the therapeutic range when daily doses ranging from 2 to 8 mg once daily were administered (Figure 3). On the other hand, patients co-administered with EIAEDs had lower trough plasma concentrations (Table 3). The highest rates (>90%) of trough plasma concentrations within the therapeutic range were observed with daily doses up to 8 mg once daily (Figure 3). Since the efficacy and tolerability of perampanel is directly related with its systemic exposure [25], it is interesting to comment on the study carried out by Gidal et al. [52]. Accordingly, the magnitude of the therapeutic efficacy of perampanel is influenced by concomitant therapy. Therapeutic response was significantly greater in patients not taking EIAEDs and receiving 8 and 12 mg/daily. The occurrence of adverse events was also greater in these patients, leading to discontinuation. In contrast, the co-administration of EIAEDs reduced the incidence of adverse events, which is consistent with the reduction in exposure caused by these drugs.

Currently, there are no recommendations for a dosing adjustment in patients co-prescribed with EIAEDs [24]. Therefore, aiming to explore the most appropriate dosing regimens of perampanel in patients taking EIAEDs, a set of simulations was herein tested for twice daily administrations (Table 4 and Figure 4). We demonstrated that patients taking EIAEDs are highly probable to obtain perampanel trough plasma concentrations within the therapeutic range if under a twice daily dosing regimen, rather than if administered with once daily dose regimen. Regarding patients not administered with EIAEDs, BMI had a greater effect, mainly when higher doses (10 to 12 mg/daily) were administered, while the greater effect of BMI in co-administered patients was found when doses ranged from 2 to 8 mg/daily (Table 3 and Figure 3). Interestingly, in patients not administered with EIAEDs and taking doses ranging from 2 to 4 mg/day, the percentage of trough plasma concentrations within the therapeutic range increased with BMI. Nonetheless, this percentage decreased when the daily drug dose ranged from 6 to 12 mg. In patients taking EIAEDs, the percentage of trough plasma concentrations within the therapeutic range increased with BMI, independently of daily perampanel dose. 

This study presented some limitations, namely the relatively small number of patients that did not allow the performance of an external validation. Moreover, the variety and complexity of concomitant antiepileptic therapy hampered the comparison between groups of patients taking perampanel and each AED. Nonetheless, the pharmacokinetic data enabled an accurate estimation of all model parameters (Table 2).

## 5. Conclusions

A population pharmacokinetic model of perampanel was successfully developed for patients diagnosed with refractory epilepsy. BMI and the co-prescription of EIAEDs were identified as covariates that significantly affect the V and CL of perampanel, respectively.

The model was able to predict pharmacokinetic parameters and subsequently the plasma concentrations of perampanel with accuracy and precision. Thus, it could be applied in clinical practice to individualize the dosing regimens of perampanel, either through an a priori model-informed strategy or an a posteriori Bayesian adjustment. 

To achieve plasma concentrations within the therapeutic range, maintenance doses ranging from 4 to 6 mg once daily are recommended for patients not co-administered with perampanel and EIAEDs. On the other hand, maintenance daily doses ranging from 4 to 6 mg twice daily are recommended for patients taking EIAEDs.

These findings highlight the importance of TDM of new generation AEDs and underline the value of model-informed dosing to personalize AED treatment. Due to its pharmacokinetic properties and narrow therapeutic window, TDM is recommended for perampanel, especially in patients also treated with other drugs that induce its metabolism.

## Figures and Tables

**Table 1:** Demographic and clinical characteristics of patients included in the study and their AED treatments.

Study Feature	Property Value
**Patients,** n	44
**Sex, *n* (%)**	
Males	27 (61.40%)
Females	17 (38.60%)
**Age** (years)	33.00 (19.00–76.00)
**Weight** (kg)	77.50 (45.00–99.00)
**Height** (cm)	168.50 (155.00–194.00)
**BSA** (m^2^)	1.88 (1.48–2.27)
**BMI** (kg/m^2^)	25.14 (15.76–36.20)
**Albumin** (g/dL)	4.40 (3.70–5.20)
**Total proteins** (g/dL)	7.00 (6.20–8.30)
**Alanine aminotransferase** (U/L)	20.00 (8.00–12.00)
**Alkaline phosphatase** (U/L)	71.00 (26.00–230.00)
**Aspartate aminotransferase** (U/L)	17.00 (12.00–73.00)
**Gamma-glutamyl transferase** (U/L)	36.50 (10.00–694.00)
**Lactate dehydrogenase** (U/L)	197.50 (138.00–260.00)
**Total bilirubin** (mg/dL)	0.45 (0.20–2.00)
**eGFR** (mL/min/1.73 m^2^)	110.63 (49.31–134.90)
**CRP (mg/dL)**	
**<0.5**	37 (84.0%)
**0.6–1.0**	3 (6.8.0%)
**1.1–3.0**	3 (6.8.0%)
**Daily dose, *n* (%)**	
2 mg	1 (2.30%)
4 mg	14 (31.80%)
6 mg	12 (27.30%)
8 mg	11 (25.00%)
10 mg	6 (13.60%)
**Co-administered AEDs per patient, *n* (%)**	
0	1 (2.30%)
1	9 (20.50%)
2	16 (36.40%)
3	13 (29.50%)
4	5 (11.30%)
**Concomitant AEDs, *n* (%)**	
Carbamazepine	15 (34.10%)
Clobazam	11 (25.00%)
Eslicarbazepine acetate	10 (22.70%)
Lacosamide	5 (11.40%)
Lamotrigine	5 (11.40%)
Levetiracetam	28 (63.60%)
Oxcarbazepine	2 (4.50%)
Phenobarbital	3 (6.80%)
Phenytoin	2 (4.50%)
Topiramate	5 (11.40%)
Valproic acid	8 (18.20%)
Zonisamide	6 (13.60%)

AED, antiepileptic drug; BMI, body mass index; BSA, body surface area; CRP, C-reactive protein; eGFR, estimated glomerular filtration rate. Results are expressed as relative and absolute frequencies or median and range.

**Table 2:** Population parameter estimates for the base and final models and bootstrap results.

Parameter	Base Model		Final Model		Bootstrap		Bias (%)
	Estimate	RSE (%)	Estimate	RSE (%)	Median	95%CI	
**TVCL** (L/h)	0.672	9.30	0.419	5.56	0.417	0.372–0.470	0.45
**TVV** (L)	33.10	10.36	29.50	6.41	29.49	23.29–32.31	0.04
**IND_CL_**	-	-	2.76	9.89	2.79	2.21–3.66	−1.27
**BMI_V_**	-	-	2.12	1.37	2.02	1.38–2.87	4.63
**IPV_C_**_L_ (%)	58.57	16.62	30.82	16.19	29.73	23.96–35.83	7.00
**RE_proportional_** (%)	9.03	20.10	6.44	24.81	6.16	3.95–7.80	8.43

BMI_V_, body mass index effect on volume of distribution; IPV_CL_, interpatient variability of clearance; IND_CL_, EIAED effect on clearance; RE_proportional_, proportional residual error; RSE, relative standard error; TVCL, typical value of clearance; TVV, typical value of volume of distribution. 95% CI; 95% confidence interval.

**Table 3:** Mean steady-state trough plasma concentration (mg/L) from simulations of different daily doses of perampanel according to body mass index (BMI) and the co-administration of EIAEDs. Concentrations within the therapeutic range of perampanel (0.1–1.0 mg/L) are shaded in grey.

Daily Dose	Without EIAEDs				With EIAEDs			
	BMI 18.5 kg/m^2^	BMI 22.5 kg/m^2^	BMI 27.5 kg/m^2^	BMI 32.5 kg/m^2^	BMI 18.5 kg/m^2^	BMI 22.5 kg/m^2^	BMI 27.5 kg/m^2^	BMI 32.5 kg/m^2^
2 mg	0.151	0.170	0.182	0.193	0.030 ^#^	0.041 ^#^	0.051 ^#^	0.058 ^#^
4 mg	0.305	0.340	0.368	0.376	0.059 ^#^	0.084 ^#^	0.103	0.115
6 mg	0.447	0.508	0.550	0.567	0.091 ^#^	0.126	0.152	0.174
8 mg	0.583	0.681	0.741	0.765	0.118	0.163	0.210	0.234
10 mg	0.764	0.847	0.909	0.941	0.144	0.208	0.260	0.287
12 mg	0.908	1.015 ^##^	1.075 ^##^	1.145 ^##^	0.178	0.246	0.309	0.347

^#^ Mean steady-state trough plasma concentration below the therapeutic range (<0.1 mg/L); ^##^ Mean steady-state trough plasma concentration above the therapeutic range (>1.0 mg/L).

**Table 4:** Mean steady-state trough plasma concentration (mg/L) from simulations of different twice-daily doses of perampanel according to BMI in patients co-administered with EIAEDs. The concentrations within the therapeutic range of perampanel (0.1–1.0 mg/L) are shaded in grey.

Daily Dose	With EIAEDs			
	BMI 18.5 kg/m^2^	BMI 22.5 kg/m^2^	BMI 27.5 kg/m^2^	BMI 32.5 kg/m^2^
2 mg	0.048 ^#^	0.055 ^#^	0.061 ^#^	0.066 ^#^
4 mg	0.097 ^#^	0.116	0.124	0.131
6 mg	0.146	0.171	0.188	0.197
8 mg	0.189	0.228	0.257	0.268
10 mg	0.239	0.276	0.309	0.331
12 mg	0.288	0.340	0.370	0.402

^#^ Mean steady-state trough plasma concentration below the therapeutic range (<0.1 mg/L).

## Data Availability

Not applicable.

## Supplementary Materials

The following supporting information can be downloaded at: https://www.mdpi.com/article/10.3390/pharmaceutics15061704/s1, Table S1: Demographic and pharmacotherapeutic characteristics of the patients included in the study; Table S2: Covariates tested for volume of distribution; Table S3: Covariates tested for clearance; Table S4: Antiepileptic drugs tested for clearance; Table S5: Covariates tested for final model. At bold is the final model.
