# Supplementary material for: Population Pharmacokinetic Analysis of Perampanel in Portuguese Patients Diagnosed with Refractory Epilepsy
Source: Pharmaceutics. 2023 Jun 10;15(6):1704. doi: 10.3390/pharmaceutics15061704 (PMC10302681; doi:10.3390/pharmaceutics15061704)
Supplement: Supplementary file 1 [file pharmaceutics-15-01704-s001.zip › pharmaceutics-2421771-supplementary.pdf]

Supplementary Materials: Population Pharmacokinetic Analysis of Perampanel in Portuguese Patients Diagnosed with Refractory Epilepsy

Rui Silva, Helena Colom, Joana Bicker, Anabela Almeida, Ana Silva, Francisco Sales, Isabel Santana, Amílcar Falcão and Ana Fortuna

Table S1 – Demographic and pharmacotherapeutic characteristics of the patients included in the study.

Patient	Sex	Age (years)	BW (kg)	HT (cm)	BSA (m²)	BMI (kg/m²)	Daily dose of perampanel (mg)	Co-prescribed ASD
1	Male	26	76	182	1.97	22.94	4	CLB, LEV, ZNS
2	Male	54	70	167	1.79	25.10	4	CBZ
3	Male	49	87	171	1.99	29.75	4	LEV
4	Male	26	45	168	1.49	15.94	4	LEV
5	Male	21	48	160	1.48	18.75	4	VA, CBZ, CLB, ZNS
6	Male	20	78	176	1.94	25.18	6	ESL, LEV
7	Female	57	55	167	1.61	19.72	6	CBZ, LEV
8	Male	20	72	180	1.91	22.22	4	VA, LEV
9	Female	23	83	166	1.91	30.12	6	CLB, LEV
10	Male	39	98	175	2.13	32.00	4	VA, LEV
11	Male	26	79	178	1.97	24.93	10	CLB, LAC, LEV
12	Female	21	57.5	160	1.59	22.46	8	CLB, LEV, OXC
13	Male	47	79	179	1.98	24.66	2	VA, ESL, LAC, LEV
14	Male	38	90	178	2.08	28.41	6	ESL, LEV
15	Male	76	80	175	1.96	26.12	4	LEV
16	Male	46	84	183	2.06	25.08	10	CBZ, LAC
17	Male	53	99	185	2.23	28.93	10	CBZ, CLB, LEV
18	Female	21	65	168	1.74	23.03	8	VA
19	Male	52	59	169	1.68	20.66	8	ESL, LTG
20	Female	26	95	162	1.99	36.20	10	CBZ, CLB, LAC, LEV
21	Male	25	85	188	2.11	24.05	8	CBZ, LTG
22	Male	27	84	181	2.05	25.64	8	ESL, PB, PHT, LEV
23	Male	30	59	163	1.63	22.21	8	LEV
24	Female	41	77	162	1.82	29.34	6	VA, LEV, TPM

25	Female	32	75	167	1.84	26.89	4	TPM
26	Male	55	97	169	2.07	33.96	8	CBZ
27	Female	54	62	158	1.63	24.84	8	CBZ, LAC, LEV
28	Female	43	69	158	1.71	27.64	6	OXC, TPM
29	Male	28	45	169	1.49	15.76	6	LEV
30	Female	44	80	155	1.79	33.30	6	CBZ, LEV, TPM
31	Female	23	55	176	1.68	17.76	6	LEV, ZNS
32	Female	23	53	158	1.52	21.23	10	CLB, LTG
33	Male	44	90	170	2.01	31.14	4	CBZ, ESL, LEV
34	Male	31	60	160	1.62	23.44	6	VA, LEV
35	Female	42	58	163	1.62	21.83	4	CBZ, CLB, ZNS
36	Male	56	94	169	2.04	32.91	8	CBZ, ZNS
37	Female	25	93.5	164	1.99	34.76	6	ESL, LEV
38	Female	34	65	164	1.71	24.17	4	-
39	Male	53	61	163	1.66	22.96	8	ESL, LTG
40	Male	64	85	175	2.01	27.76	4	CBZ, PB, LEV
41	Male	19	95	194	2.27	25.24	10	CLB, ESL, ZNS
42	Female	67	82	157	1.83	33.27	6	VA, LEV, TPM
43	Female	23	63	155	1.62	26.22	4	CBZ, CLB, PHT, LTG
44	Male	27	90	180	2.10	27.78	8	ESL, PB, LEV

BW, Body weight; BMI, body mass index; BSA, body surface area; CBZ, carbamazepine; CLB, clobazam; ESL, eslicarbazepine acetate; HT, height; LAC, lacosamide; LEV, levetiracetam; LTG, lamotrigine; TPM, topiramate; OXC, oxcarbazepine; PB, phenobarbital; PHT, phenytoin; VA, valproic acid, ZNS, zonisamide.

Table S2 – Covariates tested for volume of distribution.

Model	Covariates	Parameters	OBJ	ΔOBJ
Base	TVCL=THETA(1)<br>TVV=THETA(2)	THETA(1)=0.672<br>THETA(2)=33.10	765.384	-
1	TVCL=THETA(1)<br>TVV=THETA(2)*(THETA(3)*BW)	THETA(1)=0.666<br>THETA(2)=3.84<br>THETA(3)=0.107	753.456	ΔOBJ_{1-0}= -11.928
2	TVCL=THETA(1)<br>TVV=THETA(2)*(THETA(3)*BSA)	THETA(1)=0.669<br>THETA(2)=24.60<br>THETA(3)=0.687	760.205	ΔOBJ_{2-0}= -5.179
3	TVCL=THETA(1)<br>TVV=THETA(2)*(THETA(3)*BMI)	THETA(1)=.668<br>THETA(2)=6.61<br>THETA(3)=0.184	754.178	ΔOBJ_{3-0}= -11.206
4	TVCL=THETA(1)<br>TVV=THETA(2)*((BW/77.5)**THETA(3))	THETA(1)=0.663<br>THETA(2)=32.00<br>THETA(3)=1.55	750.765	ΔOBJ_{4-0}= -14.619
5	TVCL=THETA(1)<br>TVV=THETA(2)*((BSA/1.9)**THETA(3))	THETA(1)=0.666<br>THETA(2)=32.00<br>THETA(3)=1.99	758.045	ΔOBJ_{5-0}= -7.339
6	TVCL=THETA(1)<br>TVV=THETA(2)*((BMI/25.1)**THETA(3))	THETA(1)=0.665<br>THETA(2)=29.60<br>THETA(3)=2.07	747.792	ΔOBJ_{6-0}= -17.592
7	TVCL=THETA(1)<br>TVV=THETA(2)*(THETA(3)**SEX)	THETA(1)=0.672<br>THETA(2)=33.40<br>THETA(3)=0.971	765.363	ΔOBJ_{7-0}= -0.021
8	TVCL=THETA(1)<br>TVV=THETA(2)*(THETA(3)*TP)	THETA(1)=0.671<br>THETA(2)=12.80<br>THETA(3)=0.357	764.979	ΔOBJ_{8-0}= -0.405
9	TVCL=THETA(1)<br>TVV=THETA(2)*((TP/7.0)**THETA(3))	THETA(1)=0.671<br>THETA(2)=32.00<br>THETA(3)=0.942	764.977	ΔOBJ_{9-0}= -0.407
10	TVCL=THETA(1)<br>TVV=THETA(2)*(THETA(3)*ALB)	THETA(1)=0.671<br>THETA(2)=16.20	766.254	ΔOBJ_{10-0}= +0.87

		THETA(3)=0.451		
11	TVCL=THETA(1)<br>TVV=THETA(2)*((ALB/4.4)**THETA(3))	THETA(1)=0.672<br>THETA(2)=33.10<br>THETA(3)=0.008	765.386	ΔOBJ_{11-0}= +0.002
12	TVCL=THETA(1)<br>TVV=THETA(2)*((AGE/33)**THETA(3))	THETA(1)=0.672<br>THETA(2)=33.10<br>THETA(3)=0.038	765.364	ΔOBJ_{12-0}= -0.02

ALB, serum albumin; BW, Body weight; BMI, body mass index; BSA, body surface area; OBJ, objective function value; TP, total proteins; TVCL, typical value of clearance; TVV, typical value of volume of distribution.

Table S3 – Covariates tested for clearance.

Model	Covariates	Parameters	OBJ	ΔOBJ	IPV
Base (0)	TVCL=THETA(1)<br>TVV=THETA(2)	THETA(1)=0.672<br>THETA(2)=33.10	765.384	-	58.57%
1	TVCL=THETA(1)*(THETA(3)*BW)<br>TVV=THETA(2)	THETA(1)=0.032<br>THETA(2)=33.30<br>THETA(2)=0.291	764.030	$\Delta OBJ_{1\text{-}0}$= -1.354	57.71%<br>(-0.86%)
2	TVCL=THETA(1)*(THETA(3)*BSA)<br>TVV=THETA(2)	THETA(1)=0.502<br>THETA(2)=33.20<br>THETA(2)=0.730	763.392	$\Delta OBJ_{2\text{-}0}$= -1.992	57.27%<br>(-1.30%)
3	TVCL=THETA(1)*(THETA(3)*BMI)<br>TVV=THETA(2)	THETA(1)=0.136<br>THETA(2)=33.30<br>THETA(2)=0.195	764.193	$\Delta OBJ_{3\text{-}0}$= -1.191	57.79%<br>(-0.78%)
4	TVCL=THETA(1)*((BW/77.5)**THETA(3))<br>TVV=THETA(2)	THETA(1)=0.699<br>THETA(2)=33.20<br>THETA(2)=0.611	763.079	$\Delta OBJ_{4\text{-}0}$= -2.305	57.01%<br>(-1.56%)
5	TVCL=THETA(1)*((BSA/1.9)**THETA(3))<br>TVV=THETA(2)	THETA(1)=0.698<br>THETA(2)=33.20<br>THETA(2)=1.070	763.383	$\Delta OBJ_{5\text{-}0}$= -2.001	57.27%<br>(-1.30%)
6	TVCL=THETA(1)*((BMI/25.1)**THETA(3))<br>TVV=THETA(2)	THETA(1)=0.668<br>THETA(2)=33.10<br>THETA(2)=0.623	763.486	$\Delta OBJ_{6\text{-}0}$= -1.898	57.27%<br>(-1.30%)
7	TVCL=THETA(1) *(THETA(3)**SEX)<br>TVV=THETA(2)	THETA(1)=0.684<br>THETA(2)=33.10<br>THETA(2)=0.954	765.318	$\Delta OBJ_{7\text{-}0}$= -0.066	58.57%<br>(-0%)
8	TVCL=THETA(1)*((LDH/197.5)**THETA(3))<br>TVV=THETA(2)	THETA(1)=0.673<br>THETA(2)=33.10<br>THETA(2)=0.183	765.278	$\Delta OBJ_{8\text{-}0}$= -0.106	58.48%<br>(-0.09%)
9	TVCL=THETA(1)*((AST/17)**THETA(3))<br>TVV=THETA(2)	THETA(1)=0.672<br>THETA(2)=33.10<br>THETA(2)=0.008	765.436	$\Delta OBJ_{9\text{-}0}$= -0.052	58.65%<br>(+0.08%)
10	TVCL=THETA(1)*((ALT/20)**THETA(3))<br>TVV=THETA(2)	THETA(1)=0.675<br>THETA(2)=33.10	765.005	$\Delta OBJ_{10\text{-}0}$= -0.379	51.31%<br>(-7.26%)

		THETA(2)=0.103			
11	TVCL=THETA(1)*((AP/71)**THETA(3))<br>TVV=THETA(2)	THETA(1)=0.665<br>THETA(2)=33.00<br>THETA(2)=0.251	764.098	ΔOBJ$_{11\text{-}0}$= -1.286	57.71%<br>(-0.86%)
12	TVCL=THETA(1)*((GGT/36.5)**THETA(3))<br>TVV=THETA(2)	THETA(1)=0.689<br>THETA(2)=33.10<br>THETA(2)=0.297	758.381	ΔOBJ$_{12\text{-}0}$= -7.003	54.04%<br>(-4.53%)
13	TVCL=THETA(1)*((TB/0.45)**THETA(3))<br>TVV=THETA(2)	THETA(1)=0.672<br>THETA(2)=33.10<br>THETA(2)=0.008	765.565	ΔOBJ$_{13\text{-}0}$= +0.181	58.74%<br>(+0.17%)
14	TVCL=THETA(1)*((TP/7.0)**THETA(3))<br>TVV=THETA(2)	THETA(1)=0.664<br>THETA(2)=33.10<br>THETA(2)=1.24	764.793	ΔOBJ$_{14\text{-}0}$= -0.591	52.22%<br>(-6.35%)
15	TVCL=THETA(1)*((ALB/4.4)**THETA(3))<br>TVV=THETA(2)	THETA(1)=0.671<br>THETA(2)=33.10<br>THETA(2)=0.344	765.295	ΔOBJ$_{15\text{-}0}$= -0.089	58.48%<br>(-0.09%)
16	TVCL=THETA(1)<br>TVV=THETA(2)*((AGE/33)**THETA(3))	THETA(1)=0.665<br>THETA(2)=33.10<br>THETA(3)=0.194	764.647	ΔOBJ$_{11\text{-}0}$= -0.737	58.05%<br>(-0.52%)
17	TVCL=THETA(1)<br>TVV=THETA(2)*(THETA(3)*GFR)	THETA(1)=0.066<br>THETA(2)=33.10<br>THETA(3)=0.095	765.843	ΔOBJ$_{17\text{-}0}$= +0.459	58.91%<br>(+0.34%)
18	TVCL=THETA(1)<br>TVV=THETA(2)*(THETA(3)*(GFR/110.6))	THETA(1)=0.690<br>THETA(2)=33.10<br>THETA(3)=1.000	765.843	ΔOBJ$_{18\text{-}0}$= +0.459	58.91%<br>(+0.34%)
19	TVCL=THETA(1)<br>TVV=THETA(2)*((GFR/110.6)**THETA(3))	THETA(1)=0.681<br>THETA(2)=33.10<br>THETA(3)=0.437	764.675	ΔOBJ$_{19\text{-}0}$= -0.709	58.14%<br>(-0.43%)
20	TVCL=THETA(1)<br>TVV=THETA(2)*(THETA(3)**(GFR/110.6))	THETA(1)=0.408<br>THETA(2)=33.10<br>THETA(3)=1.66	764.730	ΔOBJ$_{20\text{-}0}$= -0.654	58.14%<br>(-0.43%)

ALB, serum albumin; ALT, alanine aminotransferase; AP, alkaline phosphatase; AST, aspartate aminotransferase; BW, Body weight; BMI, body mass index; BSA, body surface area; GFR, glomerular filtration rate; GGT, gamma-glutamyl transferase; IPV, interpatient variability; LDH, lactate dehydrogenase; OBJ, objective function value; TB, total bilirubin; TP, total proteins; TVCL, typical value of clearance; TVV, typical value of volume of distribution.

Table S4 – Antiseizure drugs tested for clearance.

Model	Covariates	Parameters	OBJ	ΔOBJ	IPV
Base (0)	TVCL=THETA(1)<br>TVV=THETA(2)	THETA(1)=0.672<br>THETA(2)=33.10	765.384	-	58.57%
1	TVCL=THETA(1)*(THETA(3)**CBZ)<br>TVV=THETA(2)	THETA(1)=0.961<br>THETA(2)=33.00<br>THETA(2)=0.498	758.814	$\Delta OBJ_{1\text{-}0}$= -6.57	54.31%<br>(-4.26%)
2	TVCL=THETA(1)*(THETA(3)**OXC)<br>TVV=THETA(2)	THETA(1)=0.656<br>THETA(2)=33.10<br>THETA(2)=1.24	764.790	$\Delta OBJ_{2\text{-}0}$= -0.594	58.22%<br>(-0.35%)
3	TVCL=THETA(1)*(THETA(3)**PB)<br>TVV=THETA(2)	THETA(1)=0.666<br>THETA(2)=33.10<br>THETA(2)=1.04	765.351	$\Delta OBJ_{3\text{-}0}$= -0.033	58.57%<br>(-0%)
4	TVCL=THETA(1)*(THETA(3)**PHT)<br>TVV=THETA(2)	THETA(1)=0.641<br>THETA(2)=33.10<br>THETA(2)=1.99	761.397	$\Delta OBJ_{4\text{-}0}$= -3.987	55.95%<br>(-2.62%)
5	TVCL=THETA(1)*(THETA(3)**CLB)<br>TVV=THETA(2)	THETA(1)=0.494<br>THETA(2)=32.80<br>THETA(2)=2.47	732.405	$\Delta OBJ_{5\text{-}0}$= -32.979	40.00%<br>(-18.57%)
6	TVCL=THETA(1)*(THETA(3)**ESL)<br>TVV=THETA(2)	THETA(1)=0.611<br>THETA(2)=33.00<br>THETA(2)=1.46	761.848	$\Delta OBJ_{6\text{-}0}$= -3.536	56.30%<br>(-2.27%)
7	TVCL=THETA(1)*(THETA(3)**LAC)<br>TVV=THETA(2)	THETA(1)=0.642<br>THETA(2)=33.30<br>THETA(2)=2.76	759.278	$\Delta OBJ_{7\text{-}0}$= -6.106	54.59%<br>(-3.98%)
8	TVCL=THETA(1)*(THETA(3)**LEV)<br>TVV=THETA(2)	THETA(1)=0.663<br>THETA(2)=33.10<br>THETA(2)=1.13	765.180	$\Delta OBJ_{8\text{-}0}$= -0.204	58.48%<br>(-0.09%)
9	TVCL=THETA(1)*(THETA(3)**LTG)<br>TVV=THETA(2)	THETA(1)=0.813<br>THETA(2)=33.10<br>THETA(2)=0.742	762.686	$\Delta OBJ_{9\text{-}0}$= -2.698	56.83%<br>(-1.74%)
10	TVCL=THETA(1)*(THETA(3)**TPM)<br>TVV=THETA(2)	THETA(1)=0.670<br>THETA(2)=33.10	765.359	$\Delta OBJ_{10\text{-}0}$= -0.025	58.57%<br>(-0%)

		THETA(2)=1.07			
11	TVCL=THETA(1)*(THETA(3)**ZNS)<br>TVV=THETA(2)	THETA(1)=0.685<br>THETA(2)=33.10<br>THETA(2)=0.852	765.056	ΔOBJ_{11-0}= -0.328	58.31%<br>(-0.26%)
12	TVCL=THETA(1)*(THETA(3)**VA)<br>TVV=THETA(2)	THETA(1)=0.715<br>THETA(2)=33.10<br>THETA(2)=0.710	763.133	ΔOBJ_{12-0}= -2.251	57.09%<br>(-1.48%)
13	TVCL=THETA(1)*(THETA(3)**IND)<br>TVV=THETA(2)	THETA(1)=0.427<br>THETA(2)=33.30<br>THETA(2)=2.71	711.125	ΔOBJ_{13-0}= -54.259	31.09%<br>(-27.48%)

CBZ, carbamazepine; CLB, clobazam; ESL, eslicarbazepine acetate; IND, enzyme-inducing antiseizure drugs; IPV, between-patient variability; LAC, lacosamide; LEV, levetiracetam; LTG, lamotrigine; TPM, topiramate; OBJ, objective funtion value;OXC, oxcarbazepine; PB, phenobarbital; PHT, phenytoin; TVCL, typical value of clearance; TVV, typical value of volume of distribution; VA, valproic acid, ZNS, zonisamide.

Table S5 – Covariates tested for final model. At bold is the final model.

Model	Covariates	Parameters	OBJ	ΔOBJ	IPV
Base (0)	TVCL=THETA(1)<br>TVV=THETA(2)	THETA(1)=0.672<br>THETA(2)=33.10	765.384	-	58.57%
1	TVCL=THETA(1)*(THETA(3)**IND)<br>TVV=THETA(2)	THETA(1)=0.427<br>THETA(2)=33.30<br>THETA(3)=2.71	711.125	$\Delta OBJ_{1\text{-}0}$= -54.259	31.09%<br>(-27.48%)
2	TVCL=THETA(1)*(THETA(3)**IND)*(THETA(4)**CLB)<br>TVV=THETA(2)	THETA(1)=0.417<br>THETA(2)=33.20<br>THETA(3)=2.64<br>THETA(4)=1.15	709.554	$\Delta OBJ_{2\text{-}1}$= -1.571	30.56%<br>(-28.01%)
**3**	**TVCL=THETA(1)*(THETA(3)**IND)**<br>**TVV=THETA(2)*((BMI/25.1)**THETA(4))**	**THETA(1)=0.419**<br>**THETA(2)=29.50**<br>**THETA(3)=2.76**<br>**THETA(4)=2.12**	**691.160**	**$\Delta OBJ_{3\text{-}1}$= -19.965**	**30.82%**<br>**(-27.75%)**
4	TVCL=THETA(1)*(THETA(3)**IND)*((GGT/36.5)**THETA(5))<br>TVV=THETA(2)*((BMI/25.1)**THETA(4))	THETA(1)=0.427<br>THETA(2)=29.50<br>THETA(3)=2.67<br>THETA(4)=2.13<br>THETA(5)=0.057	690.454	$\Delta OBJ_{4\text{-}3}$= -0.706	30.58%<br>(-27.99%)
5	TVCL=THETA(1)*(THETA(3)**IND) *(THETA(5)**LAC)<br>TVV=THETA(2)*((BMI/25.1)**THETA(4))	THETA(1)=0.420<br>THETA(2)=29.50<br>THETA(3)=2.77<br>THETA(4)=2.11<br>THETA(5)=0.978	691.137	$\Delta OBJ_{5\text{-}3}$= -0.023	30.82%<br>(-27.75%)

BMI, body mass index; CLB, clobazam; IND, enzyme-inducing antiseizure drugs; IPV, between-patient variability; GGT, gamma-glutamyl transferase; LAC, lacosamide; OBJ, objective function value; TVCL, typical value of clearance; TVV, typical value of volume of distribution.
